# Out-of-hours admissions in patients treated with immune checkpoint inhibitors and their primary management with steroids

**DOI:** 10.1177/10781552231207271

**Published:** 2023-10-17

**Authors:** Sidra Awan, Pooja Bharucha, Luke Steventon, Helen Simpson, Tanya AHMAD, Sarah Benafif, Heather Shaw, Pinkie Chambers

**Affiliations:** 1 8964 The Centre of Medicines Optimisation Research and Education, University College London Hospitals NHS Foundation Trust, London, UK; 2 Research Department of Practice and Policy, UCL School of Pharmacy, London, UK

**Keywords:** oncology, immunotherapy, hospital admissions, irAE management, steroids

## Abstract

**Introduction:**

The incidence of immune-related adverse events (irAEs) from immune checkpoint inhibitors (ICI) is well described. However, the impact on emergency care services is not. This study investigated the incidence of irAEs out-of-hours, and the management used to mitigate symptoms and side effects.

**Methods:**

This retrospective cohort study reviewed all emergency presentations triaged by the acute oncology team between December 2021 and June 2022, between 5 pm and 9 am. Patients were identified from triage audit sheets and remaining data points were retrieved from electronic health records. Inclusion criteria included all adult patients admitted on an ICI at one tertiary centre.

**Results:**

In 7 months, 970 patients called the acute oncology helpline 11% (n = 109) of patients were on an ICI treatment. After clinical review, 78% (n = 70) resulted in hospital admissions, with length of stay cumulating to 496 bed days. 56% (n = 39) of patients delayed reporting symptoms, ranging between 12 hours and 10 days from symptom onset to seeking support. 49% (n = 34) patients received steroids to manage suspected irAEs. Dexamethasone was the most common steroid used in 71% (n = 24) of patients, and variation was found in prescribed doses.

**Conclusions:**

These results underline the urgent need to address patient and staff education on adverse effects related to ICI. Patients require a comprehensive understanding of the symptoms and importance of prompt reporting. Staff education on recognition and treatment management is needed to reduce variation in practice. Further research is needed to identify barriers in symptom reporting and focus on realtime reporting to reduce the out-of-hours burden on services.

## Introduction

Immune checkpoint inhibitors (ICI) have significantly improved patient and clinical outcomes in cancer malignancies by disrupting various inhibitory pathways, including PD-1, PDL-1 and CTLA-4.^1^ A shift in the presentation of adverse effects (AEs) has been observed due to the difference in the mechanism of action compared to cytotoxic therapies. Consequently, ICI result in immune-mediated inflammation of diverse organs with varying onset-of-action. This change in presentation requires clinicians and the multidisciplinary team to develop new skills to diagnose, manage, and educate patients successfully. The broad spectrum of affected organs requires oncology teams to develop new system approaches in managing immune-related adverse effects (irAEs) with input from endocrinology, gastroenterology, pharmacy, and other specialist teams. Consensus on patient management exists, and trust-wide policies within the tertiary centre^2^ have been developed to support optimal management of irAEs, with emphasis on early recognition to minimise the progression of irAEsreducing the need for treatment interruptions and preserving quality of life. The frequencies of treatment-related irAEs are reported in both published reviews^3,4^ and the summary of product characteristics.^5,6^ The spectrum of irAEs is broad and spans multiple body systems, differing in severity and onset depending on patient intrinsic factors and the specific ICI administered. Knowledge of the timing of the onset of suspected irAE is valuable and needs to be combined with data from diverse populations in addition to clinical trials, as the quality of irAE reporting in clinical trials may not be representative of the real-world population.^7,8^ As the use of ICI increases with widening indications, novel irAEs may arise, emphasising the need for staff and patient education with early triage to support management.

The European Society for Medical Oncology (ESMO) have published a clinical practice guideline for managing toxicities arising from immunotherapy treatment.^9^ Management includes corticosteroids (CS), guiding clinicians to use the lowest effective dose. The guideline recommends the use of methylprednisolone, prednisolone, budesonide and hydrocortisone, depending on the toxicity and Common Terminology Criteria for Adverse Events (CTCAE) grade. Other steroids, including dexamethasone, have not been described within this guideline and therefore are not recommended as initial therapy. Exceptions will apply depending on co-morbidities and other patient factors. The introduction of corticosteroid can theoretically have a detrimental effect by dampening the immune response; however, findings from studies have not supported this.^10,11^

The critical first step in the management of irAEs is recognition, supported by comprehensive education for patients, caregivers, and staff working in emergency services. Following recognition of the toxicity as immune-related, clear communication pathways for healthcare providers responsible for patient care must be established. The optimal setting for emergency oncology patients varies between hospitals; therefore, ensuring clinicians are well educated on irAEs is important, as presentations can arise many months after completion of therapy. A strategy would be to re-enforce patient education in the recognition and reporting of irAE symptoms to clinical staff, providing enough information to support a correct diagnosis of irAE. Emerging approaches within oncology include the use of patient-reported outcome measures (PROMS), with recognition of irAE symptoms by patients and healthcare professionals being central to ensuring prompt and appropriate management.

At University College London Hospital (UCLH), a tertiary cancer care centre, the current approach for symptom reporting involves contacting the acute oncology service – a specialist service to support cancer patients feeling unwell during their treatment, when concerns around irAEs arise. The majority of hospitals within the United Kingdom that provide cancer services also offer acute oncology services, for the management of both cancer and treatment-related emergencies. Patients are triaged when contacting the service using a 24-hour triage risk assessment tool developed using the UK Oncology Nurses Society (UKONS) resources. The tool is used in the UK to identify and prioritise patients who contact oncology advice lines for assessment and advice.^12^ UKONS provide recommendations for best practice, recommending telephone triage to determine the severity of the patient's symptoms and direct them either to emergency assessment or to encourage self-management. The Cancer Reform Strategy, published in 2007,^13^ is a policy that ensures patients and carers are given 24 hour contact details for their acute oncology services.

Our study investigated the volume of out-of-hours calls received from patients receiving ICI, examining the pathway of care to determine variations in practice. As part of this investigation, we wanted to identify if there were delays in symptom reporting, and whether this impacted emergency department admissions in terms of bed days used. Secondary aims included evaluating steroid management, including the choice of steroid and total cumulative dose.

## Method

A retrospective review was undertaken at UCLH from December 2021 to July 2022 of all patients on ICI treatments admitted through the acute oncology service (AOS) from the triage helpline.

### Data source

A database of all out-of-hours calls received through the triage helpline is maintained as an Excel 2010 spreadsheet (Version 14.0.7192.5000) and securely held in password protected folders within the tertiary centre system. This database contains a locally registered audit collection tool used by clinical nurse specialists and medical staff to populate all queries raised by patients from 5 pm to 9 am, following risk assessment adapted from the UKONS tool. Data includes patient demographic details: primary diagnosis, admission history, treatment history, and triage plan. We used this collection tool to identify all patients over 18 years old treated with ICI.

Further data were required to meet our objectives. A data collection tool was developed using Microsoft Excel, and additional data were manually retrieved from the electronic medical record retrospectively. Data were collected between August and September 2022 by author SA. A timeframe of 2 weeks was set for manual data collection, resulting in data from a seven-month period being collected. Additional data was retrieved for each patient, included patient demographics (age, sex, weight, height and ethnicity), patient characteristics, presenting complaint (PC), duration of PC (hours), whether healthcare providers saw patients in the preceding two weeks before presenting to the emergency department, and treatment characteristics (type of immunotherapy and number of cycles given). Data on each patient's admission to the hospital (length of stay, admission to ICU or in-patient ward, class of treatments prescribed on day 1, steroid choice, steroid dose on day 1, steroid cumulative dose, steroid de-escalation) were also collected. The CTCAE version 5.0 was used to classify the presenting complaint to the system organ class.

Data collection was verified by randomly selecting 10% of the total number of patients and manually collecting data from the electronic medical records by author LS on all fields. Both data sheets were presented to author PB who reviewed the data collection sheets, and any discrepancies between author LS and SA were reviewed independently and rectified using electronic prescribing patient records. Discrepancies were only found in treatment cycles (n = 2), clinical trial (n = 1) and length of stay (n = 1). PB was aware to escalate to authors SA, LS, and PC if any errors were identified on any of the variables affecting the primary aim and secondary aim, which would have resulted in increasing the verification sample to 20%.

Using RStudio basic, data were analysed using descriptive statistics (count, percentage, median, and range).

## Results

970 patients called out of hours seeking advice related to their symptoms, as shown in Table 1. Of these, 109 (11%) were on ICI and called between 5 pm and 9 am with suspected irAEs. After review using the assessment tool, 90 (83%) patients were advised to attend the emergency department for further investigation and medical assessment. Based on toxicities and patients’ symptoms, 67 (74%) were admitted to the hospital, and three (3%) were admitted and then escalated to intensive care within 48–96 hours. Four hundred ninety-six days were cumulatively spent in the hospital, with a median stay of 3 days per patient (range 1–36).

**Table 1.** Outcome of acute oncology triage service.

	**Number (%)**
**Acute oncology service helpline**	
Total number of calls	970 (100%)
Total number of calls related to ICI	109 (11%)
**The outcome of oncology triage risk assessment (n = 109)**	
Presenting to Emergency Department (ED)	90 (83%)
**The outcome of the ED assessment (n = 90)**	
Admitted to In-patient ward (level 1)	67 (74%)
Escalated to Intensive Care (levels 2–3)	3 (3%)
**Cumulative length of stay**	**Bed-days**
All in-patient admissions	496
Median, Range	3 (1–36)

Table 2 provides an overview of the demographics of patients included in this study. 56 (62%) patients were male, and 34 (38%) were female. The median age was 65 years (range 25–86). The most common malignancies were lung (n = 37; 41.1%) and genitourinary (GU) (n = 12; 13.3%). The median number of treatment cycles received before developing an irAE was 2 (1–24). The demographic data, including the primary tumour site, are shown in Table 2.

**Table 2.** Patient characteristics of those being treated with ICS presenting out of hours as an emergency.

	**Number, %**
**Characteristic**	
Age (median, Range)	65 (25–86)
**Biological Sex**	
Male	56 (62%)
Female	34 (38%)
**Clinical Trial**	
Yes	40 (44%)
**Treatment cycles**	
Cycles of ICI received before irAE (median, Range)	2 (1–24)
**Primary Cancer diagnosis**	
Lung	37 (41.1%)
GU	12 (13.3%)
Upper GI	11 (12.2%)
Lower GI	8 (8.9%)
Gynae	8 (8.9%)
H + N	6 (6.7%)
Breast	3 (3.3%)
Melanoma	3 (3.3%)
Sarcoma	2 (2.2%)

Abbreviations: GU: Genitourinary; Upper GI: Upper Gastrointestinal; Lower GI: Lower Gastrointestinal; H + N: Head and Neck.

The most common primary presenting complaint classified by organ class (CTCAE) were gastrointestinal (GI) disorders 35 (23%) and infections 22 (15%), as shown in Table 3. Those who experienced GI irAEs, presented with specific complaints of diarrhoea (n = 17; 49%), abdominal pain (n = 13; 37%), and colitis (n = 5; 14%). Those presenting with infection experienced pyrexia (n = 16; 73%) and sepsis (n = 6; 27%) as well as a secondary presenting complaint varying from GI, nervous system, vascular and eye disorders (n = 19; 86%).

**Table 3.** Presenting complaints classified according to the CTCAE v5.0.

**Presenting complaint- System Organ Class (CTCAE)**	**Number of complaints (n = 149)**
Gastrointestinal disorders	35 (23%)
Infections and infestations	22 (15%)
Respiratory, thoracic and mediastinal disorders	20 (13%)
Nervous system disorder	19 (13%)
Metabolism and nutrition disorders	13 (9%)
Vascular disorders	12 (8%)
Injury and procedural complications	11 (7%)
General disorders	9 (6%)
Psychiatric disorders	2 (1%)
Renal and urinary disorders	2 (1%)
Musculoskeletal and connective tissue	1 (0.7%)
Skin and subcutaneous tissue disorders	1 (0.7%)
Eye disorders	1 (0.7%)
Hepatobiliary disorder	1 (0.7%)

Table 4 shows the proportion of patients admitted within each malignancy. Thirty-one lung cancer patients (84%) were admitted, with a cumulative stay of 234 days. Of these, 16 patients (52%) experienced their symptoms longer than 12 h before calling the acute oncology service out-of-hours (ranging between 12 hours and 72 hours), and 13 lung cancer (42%) patients had no documentation in their notes regarding the duration of symptoms. The most extended duration that patients remained symptomatic for prior to escalating irAEs to healthcare providers was 10 days within upper GI patients, followed by 7 days in patients with gynaecology and head & neck cancer. The upper-GI cancer patient presenting to ED experiencing two symptoms; difficulty swallowing and pain, was subsequently administered steroids (dexamethasone) and opioids (morphine). The gynae-oncology patient experiencing colitis and hepatic pain was administered steroids, whilst the head & neck cancer patient who experienced a fall required neurosurgical intervention, resulting in intensive care admission. In total across all malignancies, 27 of 90 total patients had no documentation in their notes regarding the duration of symptoms.

**Table 4.** Length of stay (LOS) for patients admitted according to their primary cancer diagnosis.

**Disease group**	**Patients admitted to the in-patient ward**	**Total LOS (days)**	**No. of patients with irAE symptoms >12 hours**	**History of presenting complaint Duration in hours (range)**
Lung (n = 37)	31	234	16	12 −72
GU (n = 12)	12	113	7	16 −72
Upper GI (n = 11)	6	50	6	24 −240
Lower GI (n = 8)	6	24	3	24–120
Gynaecology (n = 8)	4	12	2	72–168
H + N (n = 6)	5	35	1	168
Breast (n = 3)	2	11	1	20
Melanoma (n = 3)	3	15	2	72–120
Sarcoma (n = 2)	1	2	1	24

The number of patients with presenting complaints lasting >12 hours recorded and the range of durations in hours. Abbreviations: GU: genitourinary; Upper GI: upper gastrointestinal; Lower GI: lower gastrointestinal; H + N: head and neck.

Thirty-four patients received pharmacological doses of steroids. Of these patients, 24 (71%) patients were treated with dexamethasone intravenously/orally, 5 patients (15%) were treated with methylprednisolone intravenously (1–2 mg/kg dose range), 3 patients (9%) were treated with hydrocortisone intravenously (100 mg–500 mg dose range) and 2 patients (6%) treated with prednisolone orally (1 mg/kg). The median length of stay for this cohort was 6 days (0–19 days), and 21 patients (62%) had symptoms lasting longer than 12 hours with a median of 24 hours (12–240 hours).

Dexamethasone was the most common steroid used. Four patients (17%) had documented brain metastases and seven patients (29%) with a documented drug history of its use before admission. Details of the dosing used on day 1 of admission are presented in Table 5.

**Table 5.** Dexamethasone prescribing.

	**Number (%)**
**Cumulative dose, Day 1 (n = 24)**	
1–5 mg	3 (13%)
6–10 mg	10 (42%)
16 mg–20 mg	10 (42%)
21–25 mg	1 (4%)
**Cumulative dose, total course**	
1–50 mg	13 (54%)
51–100 mg	6 (25%)
101–150 mg	2 (8%)
151–200 mg	1 (4%)
200–300 mg	2 (8%)
**Dose interval (n = 24)**	
OD	12 (52%)
BD	8 (30%)
TDS	3 (13%)
QDS	1 (4%)
**Course duration, days (n = 24)**	
1–5	14 (57%)
6–10	6 (26%)
11–15	2 (7%)
16–20	2 (7%)

Abbreviations: mg: milligrams; OD: once daily (8 am); BD: twice daily (8 am; 12 noon); TDS: three times daily (8 am, 12 noon; 4 pm), QDS, four times daily (8 am, 12 noon, 4 pm, 10 pm).

## Discussion

This retrospective study of suspected irAEs in patients treated with ICI, showed a high proportion (83%) of those seeking assistance out-of-hours are admitted in the hospital resulting in increased length of stay cumulatively. The out-of-hours period comprises 67% of the week. It poses additional challenges, including accessing patient information and meeting clinical needs, with evidence suggesting that admissions to hospitals on weekends are associated with poorer patient outcomes.^14,15^ In this study, we demonstrate that the median number of cycles of immunotherapy completed was two cycles prior to patients presenting to ED with varying symptoms of irAEs.

This study supports interventions should be targeted to improve the management pathways for irAEs. We found that 56% of patients had ongoing symptoms for more than 12 hours (ranging from 12–240 hours) before presenting to the ED out-of-hours. The range of irAEs, can, in principle, affect any tissue or organ and vary from mild to severe. This adds additional challenges in communicating, educating, and ensuring this information is retained to support prompt action if patients begin to develop irAEs in the community. In our study, patients had a time delay before sharing this information with healthcare providers. Jamieson and colleagues^7^ also identified this feature and explained this through Walter's model, which describes key time points in the diagnostic pathway, specifying where delays in help-seeking can arise. Reasons for this were not explored within our study; however, Jamieson et al. identified patients’ anxiety around treatment cessation as a contributor to delayed reporting, giving examples of minor adverse events escalating to significant events requiring hospital admission. These delays in symptom reporting are crucial to address and are particularly relevant for the 3% of our patients that were escalated to the Intensive Care Unit, leading to a stay of 1–5 days. In these three cases, one patient had no documentation of the history of their adverse events, and two experienced symptoms at home for 16 hours and 7 days, respectively. Earlier identification of these symptoms would likely have provided more management options and the opportunity to prevent symptoms from escalating. The healthcare provider-led approach at our tertiary centre regarding symptom reporting is through regular clinics, scheduled chemotherapy appointments and the acute oncology triage unit out-of-hours, as well as educating patients on using the triage advice line for symptom reporting. Basche et al.^16^ have demonstrated a different approach using patient-reported outcomes, which enabled enhanced symptom monitoring and demonstrated clinical benefits. Utilising a digital approach and supporting patients to report symptoms in real-time allowed healthcare providers to initiate appropriate interventions, which improved patient quality of life and reduced emergency department admissions and treatment interruptions. This method would likely allow for improved symptom management from the perspective of both patients and practitioners and would enable the current healthcare delivery model to evolve significantly with new technology to achieve favourable clinical and patient outcomes.

This study provided real-world data on out-of-hours admissions and presenting complaints in patients treated with ICI, demonstrating that gastrointestinal disorders were the most common, followed by infections and respiratory disorders. Identifying the most common complaint per primary diagnosis showed respiratory and mediastinal disorders were dominant in lung cancer (25.4%) (see Table 3). GI disorders were most common in upper GI, lower GI, gynaecology, and melanoma cancers (see Table 3). Focusing on GI disorders, 24 patients presented with ongoing symptoms for >24 hours (up to 168 hours). The symptoms identified included diarrhoea, dehydration and abdominal pain, which could have been managed successfully with earlier intervention and could have reduced the length of in-patient admission. Depending on the severity of symptoms, immunomodulatory treatments were initiated to treat irAEs. Within this study, 49% were treated with steroids, including methylprednisolone (MP), prednisolone, hydrocortisone and dexamethasone. Despite local and international guidelines recommending the use of MP or prednisolone in most cases, dexamethasone has not been identified as a therapeutic option for immunotherapy irAEs.^2,8^

The use of corticosteroids is integral to the symptomatic treatment of irAEs, and it is essential to note that within clinical trials, 40% of studies listed glucocorticoid administration as an exclusion criterion (>10 mg daily prednisolone)^17,18^ due to the working theory of the immunosuppressive effect counteracting the working mechanism of ICI. As a result, glucocorticoid administration is a neglected variable in immunotherapy outcomes. Prednisolone 10 mg equates to 1.5 mg of dexamethasone in glucocorticoid potency,^19^ and therefore the dosages used within our study to ameliorate irAEs should be taken into account. In addition, using glucocorticoids for other indications will result in high cumulative dosing, which, if unregulated, can counteract the positive benefits of its use. A systematic review on the combined use of steroids and ICIs in brain metastasis patients^20^ showed a significantly worse overall survival and progression-free survival, with authors identifying the need for further research on dosing, timing, and duration of steroids with ICI. In contrast, Umehara et al.^21^ demonstrated no differences in the therapeutic effect of nivolumab in the irAE sub-group post-corticosteroid administration in patients with non-small lung cancer. Further research is needed to identify if steroids obstruct the immunoreactivity of ICI at varying doses, as this study has demonstrated variation in prescribing practice.

Dexamethasone is used widely in cancer therapy and is particularly beneficial in patients with brain tumours with significant peritumoral oedema. Despite the longstanding use of dexamethasone since it was first synthesised in 1958, there have been few clinical trials to determine the optimal dose. Specific properties of this steroid (long biological half-life, low mineralocorticoid activity) have led to favourability amongst oncologists. However, significant variation exists in dosing, duration, weaning regimens and high burden of side effects (for example, insomnia, avascular necrosis, and mood changes).^9^ This study demonstrates the variation in prescribing practice and the need for education among clinical staff regarding the differences in pharmacodynamics between steroid formulations and their impact on potency. In addition, our study demonstrated variation in dose interval prescribing where dexamethasone was prescribed “QDS; four times daily” and “TDS; three times daily” despite the biological half-life of 36 h, however, dose timings were not always available retrospectively in hospital records and so were not investigated further in this study. Prescribing treatments beyond the recommended ranges increases the risk of side effects amplifying the initial presenting complaint and can contribute to prolonged hospital admissions.

The limitations of this study were that it was retrospective and therefore resulted in some missing data, limiting our analysis. Additionally, the time period chosen was during the introduction of a new electronic prescribing system, and therefore some calls received may not have been appropriately recorded. Lastly, the documentation of the grade of irAE (CTCAE grading 5.0) and the inconsistency of information recording was not double checked as it would have been if collected prospectively. Variations in practices meant we chose to omit this variable within the data collection. Despite these limitations, our work has been essential in guiding service changes at UCLH, including focusing on immunotherapy education and recruitment of additional staff to support this service.

This study showed that during the out-of-hours period, 11% of total calls received through the acute oncology service were related to ICI and the presenting symptoms of irAEs. Of the patients advised to attend the emergency department, 83% were subsequently admitted to hospital, and 56% had experienced symptoms between 12 hours and 10 days before contacting the hospital out-of-hours helpline and attending ED. The length of stay cumulated to 496 days for these 70 patients admitted to ED, and 49% were treated with steroids for their irAEs. Of these, 68% were treated with dexamethasone, a steroid not routinely used to manage irAEs.

Our service evaluation has demonstrated the importance of patient education in identifying irAEs. New approaches are needed to support real-time adverse effect reporting. This study also highlighted the need for staff education on symptom management and establishing clear communication pathways with patients as there is significant variation in steroid use for irAE management. Future investigations in this area may involve audits of current practice against the ESMO guidelines and summary of product characteristics to assess the appropriateness of steroid use in irAE management.
